# A three-way approach for protein function classification

**DOI:** 10.1371/journal.pone.0171702

**Published:** 2017-02-24

**Authors:** Hafeez Ur Rehman, Nouman Azam, JingTao Yao, Alfredo Benso

**Affiliations:** 1 Department of Computer Science, National University of Computer and Emerging Sciences, Peshawar Pakistan; 2 Department of Computer Science, University of Regina, Regina, SK S4S 0A2, Canada; 3 Department of Computer & Control Engineering, Politecnico di Torino, I-10129, Torino, Italy; Southwest University, CHINA

## Abstract

The knowledge of protein functions plays an essential role in understanding biological cells and has a significant impact on human life in areas such as personalized medicine, better crops and improved therapeutic interventions. Due to expense and inherent difficulty of biological experiments, intelligent methods are generally relied upon for automatic assignment of functions to proteins. The technological advancements in the field of biology are improving our understanding of biological processes and are regularly resulting in new features and characteristics that better describe the role of proteins. It is inevitable to neglect and overlook these anticipated features in designing more effective classification techniques. A key issue in this context, that is not being sufficiently addressed, is how to build effective classification models and approaches for protein function prediction by incorporating and taking advantage from the ever evolving biological information. In this article, we propose a three-way decision making approach which provides provisions for seeking and incorporating future information. We considered probabilistic rough sets based models such as Game-Theoretic Rough Sets (GTRS) and Information-Theoretic Rough Sets (ITRS) for inducing three-way decisions. An architecture of protein functions classification with probabilistic rough sets based three-way decisions is proposed and explained. Experiments are carried out on *Saccharomyces cerevisiae* species dataset obtained from *Uniprot* database with the corresponding functional classes extracted from the Gene Ontology (GO) database. The results indicate that as the level of biological information increases, the number of deferred cases are reduced while maintaining similar level of accuracy.

## Introduction

All living organisms are composed of cells, which are intricately arranged chemical factories that obtain matter from their environment and use this raw matter to generate copies of themselves [1]. Behind this miraculous functioning of the cells are the most important biochemical molecules called proteins. Due to their role in almost every biological activity, it is crucial to have a clear understanding of their respective functions. Moreover, the knowledge of protein functions is also essential for understanding how biological activities are performed at the molecular level. This is useful in developing personalized medicine, more effective therapeutic interventions as well as understanding biological entities as engineered systems [2–7]. On the other hand, when the number of sequenced genomes are growing, the overwhelming majority of new proteins with unknown functions continue to emerge at an exponential rate. Under these conditions, it is not feasible to manually identify and assign functions to proteins. Intelligent mechanisms are generally relied upon to automatically predict and assign functions to proteins [8–10].

Several methods have been proposed for characterization of protein functions. The early and conventional techniques were generally based on the most fundamental type of information about proteins i.e., their amino acid sequence, utilizing tools such as Basic Local Alignment Search Tool (BLAST) [11]. Sequence of a protein determines its different characteristics such as its sub-cellular localization, possible structural conformations as well as its functions [3]. Some of the prominent approaches in this category can be found in [12–14]. With the availability of data from massive high-throughput experiments, features based on different data such as genomic contextual data and Protein-Protein Interactions (PPIs) data, has also emerged. Recent and advanced computational methods utilized these and similar information in designing approaches for prediction task. For example, features based on genomic contextual data were utilized by [12, 15, 16], features based on protein-protein interaction data were used by [17–20], and features exploiting function structure relationship were reported in [21–23]. As we have access to more interesting information, we may expect more effective models and approaches for precise prediction of protein functions.

Due to technological advancements, our understanding of biological processes is improving and new features describing proteins are emerging on regular basis [3]. It is inevitable to ignore these anticipated features in designing more effective and efficient prediction techniques. An important issue that needs to be addressed in this context is how to develop effective models by incorporating and taking advantage from the ever evolving biological information that leads to new features and characteristics of proteins. This however has generally been overlooked and received little or no attention in the existing literature. A general assumption, although not explicitly stated, is that the information is being fixed (i.e., not dynamic and evolving) while developing classification approaches. This assumption may not be always useful, for instance, consider the classification of proteins whose functions may not be precisely identified due to lack of associated biological information (although we may anticipate it in future) thereby leading to compromised results. To address this issue, i.e., incorporating the anticipated future information into the predictive task, we propose a three-way decision making approach that includes a decision option of deferment. This option is exercised whenever we have inconclusive and insufficient evidence to reach confirmed or certain decisions. The deferred decision option provides provisions for incorporating future information which may be used in deciding the deferred cases. In particular, three types of decisions are used, i.e., accept, reject and deferment in order to classify functions of proteins.

There are different models for inducing three-way decisions. In this article, we investigate and examine probabilistic rough sets based three-way decision making approaches for protein functions classification [24]. The probabilistic rough sets can be used to induce three regions corresponding to a concept (represented in terms of a set), namely, positive, negative and boundary regions. The three regions lead to three-way decisions in the form of acceptance, rejection and deferment, respectively. The three regions and their respective decisions are defined and controlled by a pair of thresholds. There are different forms and models of probabilistic rough sets based on how these thresholds are obtained and interpreted. We consider two such models, i.e., Game-Theoretic Rough Sets (GTRS) [25–27] and Information-Theoretic Rough Sets (ITRS) [28]. Moreover, we examine and define five three-way approaches based on the GTRS and ITRS by employing different measures and iterative methods. To incorporate and take benefit from these three-way approaches in real applications, we propose an architecture of protein functions classification. Lastly, we evaluated the three-way approaches on the dataset of *Saccharomyces cerevisiae* species proteins which is obtained from *Uniprot* database [29], with the corresponding functional classes extracted from the well known Gene Ontology (GO) database [30]. The experimental results indicate that by increasing the level of biological information associated with proteins, the number of deferred cases can be reduced while maintaining the same level of accuracy. We comprehensively benchmark our approaches under these settings and conclude that the classification becomes more crisp as the knowledge of associated biological information matures.

The code (Python/Bash/Matlab) and data files used in this work are available as a zip file (“Protein_Functions_TWD_data_code.zip”) from http://tinyurl.com/jdpwkkq.

## Background

### Protein function classification

An important factor that impacts the performance of function prediction models is the type of biological information used to infer functional association among proteins. Until recently, many high throughput techniques have been developed to devise mechanisms leading to precise prediction of protein functions. These techniques utilize information derived from sequence similarity, protein 3D structure, phylogenetic profiles, protein complexes, PPIs, gene expression profiles [31–33]. The most prominent techniques utilize proteome-scale PPI networks that have been retrieved for several organisms including yeast and human. Protein-protein networks are graphs where each node represents a protein and edges between nodes represent an interaction. An interaction in the network is either a direct physical association between the proteins (typically retrieved via two hybrid analysis [34] or on the other hand if two interacting proteins are part of the same multi-protein complex, they are also considered as interacting proteins [35]. Thus from informatics point of view an interaction is not necessarily a direct physical association of proteins but sometimes it is mutual presence in the same protein complex depending on the experiment which reveals the interaction.

The most recent as well as renowned approaches in the field of protein function prediction use protein-protein interactions data in different ways [31–33]. A wide majority of these techniques are based on the fact that interacting proteins are likely to share common functions as they interact for an associated biological activity. Methods in this category assign annotations to protein under question, based on the functions of their neighboring proteins. The methods vary in the extent to which they employ global features of the interactome in annotating proteins, or the way they exploit the topological features of the interactome [17, 18]. In addition to that, the methods are based on quite varied underlying formulations and use well understood concepts from the fields of graph theory, graphical models, stochastic processes, probabilistic graphs or clustering [18, 19].

Another class of approaches are based on utilizing the GO structure into computational models by incorporating the semantic similarity offered by the Direct Acyclic Graph (DAG) architecture of gene ontology. The integration of multi-level gene ontology terms exploiting their relationships for protein function prediction was investigated in [2, 8, 9, 36]. These methods calculate different similarity measures by operating on GO term dependencies to define functional associations among proteins. A similar technique based on the Markov Random Field (MRF) properties of protein-protein networks, integrated the inter-species protein homolog information to construct MRF based graphs using the gene ontology terms was outlined in [8]. The authors report high precision when tested for a limited set of functional terms [8].

Another type of biological information that is frequently used for uncharacterized proteins is the number of motifs conserved in those proteins [9, 36]. Several functionally conserved proteins are found to have motifs that associate them to a particular molecular activity. For example, hypothetical protein YIL169C is conserved with Chemotaxis_Transduce_2 and T_SNARE motifs, and similar motifs in known proteins can be used to link functional information with the protein under investigation. Integrating heterogeneous information conserved across proteins of unknown function, with state of the art classification scheme may help to increase protein function prediction accuracy.

The existing computational approaches have significantly contributed in understanding and characterizing protein functions by investigating and utilizing different types of features. However, there is still a need for approaches to incorporate and integrate the ever evolving features of proteins for precise prediction of their functions. These new features, once known and available, will give better insight into biological activities thereby are expected to provide more precise characterization of proteins. In the later sections of this article, we present a three-way approach to address these issues.

### Three-way decisions

In many real life decision making scenarios involving vague and uncertain information, the three-way decision making strategy including a delay, deferment or non-commitment decision option is a better and more useful approach [37–39]. To explain this, consider the following examples: 1) How do we make a purchase decision based on information gathered from blogs, reviews, friend suggestions and experiences? 2) How do doctors make diagnosis decisions based on the presence of some symptoms and tests? 3) How do military commanders decide to carry out military actions based on intelligence information? In all these and similar decision scenarios, the decision makers are faced with two types of situations. Either they have sufficient and convincing information necessary to make a decision or they are faced with vague and incomplete information which is insufficient to make a useful decision. In the former case, the decision makers can exercise immediate and certain decisions in the form of acceptance/rejection, yes/no or true/false. In the latter case, the decision makers may not be able to make certain decisions. For instance, the diagnosis tests are inconclusive or the intelligence information is vague or incomplete. A better and more useful choice in such uncertain and doubtful situations is to delay the decisions, assuming that future information will evolve which will make the decision making more obvious and evident. Three-way decisions is essentially the same approach to decision making. We make immediate accept or reject decisions if we have convincing and sufficient evidence based on the available information. On the other hand, we make a deferment decision whenever we lack sufficient evidence.

In fact, three-way decisions has been practiced over the years across different domains, including, medical decision making, psychology, social judgment theory, management sciences and machine learning [40–44]. These application domains suggest that three-way decisions enjoy a good history from usage and application perspective, however, it is surprising to note that from theoretical perspective, it lacks a unified formal description over the years [45]. This theoretical gap was first recognized in the rough sets community. In particular, Yao introduced a general theory of three-way decisions, motivated by the rough sets based three regions [45]. The essential notion in the theory adopted from rough sets is the division of the universe into three pair-wise disjoint regions. The theory however is not restricted to rough sets and goes beyond it by considering rough set theory as one of many possible ways to construct and induce the three regions [39, 46]. Three-way decisions may be formulated based on the theories such as rough sets, interval sets, shadowed sets, approximations of fuzzy sets, a threshold approach in medical and orthopairs [47–54].

An important consideration in formulating three-way decisions is the division of the universal set into three pair-wise disjoint regions. It is recently argued that an equally important consideration is the design of effective strategies for processing the three regions [39]. The realization of these two essential components, i.e., division and processing lead to the trisecting and acting framework of three-way decisions [39].

The trisecting and acting framework explains and presents three-way decisions as a two step process. In the first step, i.e., trisecting, the universe is divided into three pair-wise disjoint regions. This means that we seek tripartition of the universe. In the second step, i.e., acting, strategies are designed for processing the three regions to obtain three-way decisions. This framework aimed at introducing three-way decisions at a more generic level. Generally, the division of the universe is carried out based on an evaluation function and a pair of thresholds. The evaluation function assign an evaluation value to each object by employing some criteria. The objects whose evaluation values are at or above a certain threshold of acceptance makes up the POS region. The objects whose evaluation values are at or below a certain threshold of rejection make up the NEG region. The objects whose evaluation values are above the rejection threshold but below the acceptance threshold make up the BND region. A specific definition of evaluation based three-way decisions based on a single evaluation function (used for evaluating both acceptance and rejection) and totally order set is given in [39].

There are many issues and challenges for building and using three-way decision models. Some of these issues include the definition and construction of evaluation functions, the definition of the domains for the evaluation functions, the determination and interpretation of acceptance and rejection levels, the measurement of the quality of the three regions, generation of predictive rules from the three regions for making decisions on new objects, descriptive rules for describing the three regions and design of strategies and actions corresponding to the three regions [39, 45]. Based on how these issues are handled and interpreted, we may have different three-way decision making models and approaches. We focus on three-way decisions with probabilistic rough sets.

## An architecture of protein function classification with three-way decisions

**Fig 1:**
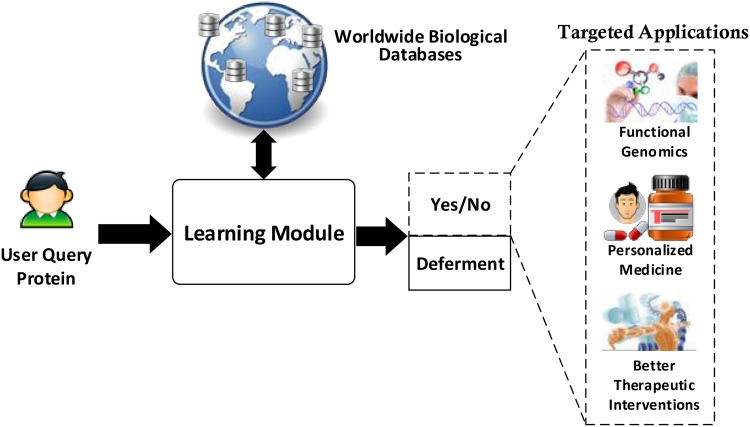
Logical view of the architecture with three-way decisions for protein function classification.

**Fig 2:**
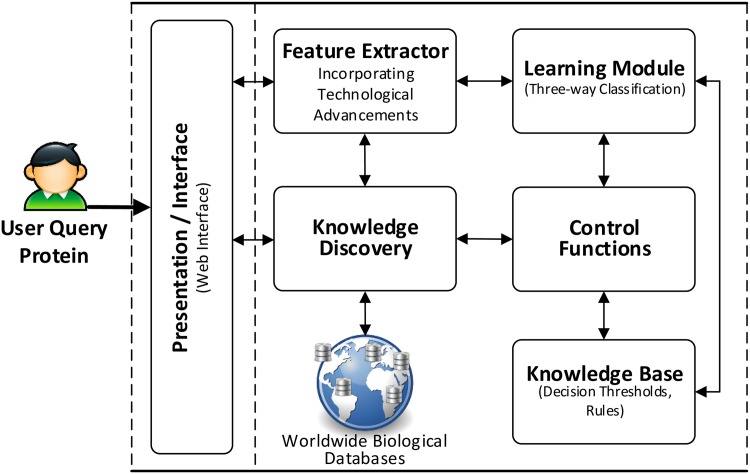
Physical view of the architecture with three-way decisions.

To make effective use of three-way decisions, we propose an architecture for supporting protein functions classification decisions. The architecture may be utilized in building systems to provide decision support capabilities for deciding protein functions. Fig 1 shows the logical view of the architecture and highlights its intended applications. The architecture supports user queries in the form of protein IDs (also called Uniprot IDs) which are mapped to functional classes by making use of three-way decisions. Fig 2 shows the physical view of the architecture along-with its various components. These components have different capabilities and functionalities ranging from supporting interaction of end user using interface to storing, collecting and manipulating the data for providing decision support.

The clients or end users will interact with the system through interfaces. Considering a typical client-server model over the Internet platform where the system is deployed over the servers and provides services by responding to client queries. The interfaces may be presented to the end users through Web browsers. The users can send their queries on proteins and see the resulting functions returned by the system. The interfaces should be carefully designed and has to be clear, complete, consistent and should provide guidance to users for correctly using the systems. In some cases, it may also provide auto correction facility. Besides interface, there are various other components at the core of the architecture namely Knowledge Discovery, Feature Extractor, Learning Module, Knowledge Base and Control Functions. We now explains each of them briefly with their intended functionalities.

**Knowledge Discovery/Information Retrieval:** The Knowledge Discovery module interacts with both the worldwide biological databases and feature extractor module. As new features are evolving, the feature extractor module may require different type of biological information to compute feature values. On one hand, it will provide querying and searching facilities for extracting information from relevant biological databases and on the other side, it is responsible for passing them to the feature extractor module.

**Feature Extractor:** The features describing proteins are computed based on relevant data extracted from biological databases which are spread around the world. The feature extractor module request or query the information retrieval component for providing relevant information necessary for computing a feature value. The information retrieval component extracts the required information from the world wide biological databases. For example, for getting one of the features namely, protein interaction networks (PIN), this module will ask for relevant information, i.e., number of interactors corresponding to a protein. The information retrieval identifies and searches the relevant databases such as STRING and IntAct databases and will pass the respective information to the feature extractor. The feature extractor module is then responsible for further processing in order to calculate the feature value, e.g. the number of interactors present in both the databases. As new features become available due to technological advancements, the feature extractor module will ask for new type of information from the information retrieval component and do the relevant computation and processing to calculate feature values. In section **Data Preparation**, we describe different types of features that have evolved over the time and explain the types of data that is required to computed them.

**Learning Module:** The learning module interacts with the feature extractor and knowledge base. This module will incorporate intelligent techniques to make useful inferences from the data to reach effective classification decisions. In this article, we suggest a classification mechanism based on three-way decisions as one of the possibility. An important output of the learning module will be a set of functions that are being performed by a protein.

**Knowledge Base:** The knowledge base contains necessary information that is learned and made available by the learning module. The information, such as, decision thresholds and rules for classifying proteins may be stored in the knowledge base for future use.

**Control Functions:** This module is included to ensure security and protect the system from attacks and unauthorized usage. It should provide functionalities such as access rights and permissions.

## Realization of rough sets based three-way decisions

Three-way decisions is a better and useful choice in applications with evolving information. In this section, we explain this phenomena using rough sets based three-way decisions. For the sake of completion, we review the main notions of rough sets.

### Three-way decisions using rough sets

Three-way decisions using rough sets are defined by considering an information table *S* which is defined as a tuple.

$$\begin{array}{c} S=(U,At,\left\{V_{a}|a\in At\right\},\left\{I_{a}|a\in At\right\}), \end{array}$$ (1)

where *U* is a finite set of objects also known as the universe, *At* is a finite set of attributes, *V*_*a*_ is the domain of attribute *a* ∈ *At* and *I*_*a*_ is an information function which provides a mapping from *U* → *V*_*a*_. In particular, the information function *I*_*a*_ assigns to each object *x* ∈ *U* a value in *V*_*a*_ i.e., *I*_*a*_(*x*) ∈ *V*_*a*_. A major concern in rough set theory is how to discern objects. The equivalence relation defined on *U* is used for this purpose. For a set of attributes *A* ⊂ *At*, the equivalence relation, namely, *E*_*A*_ is defined as,

$$\begin{array}{c} E_{A}=\left\{\left(x,y\right)\in U\times U|\forall a\in A,I_{a}\left(x\right)=I_{a}\left(y\right)\right\}. \end{array}$$ (2)

This means that any two objects *x* and *y* in *U* are equivalent or in other words indiscernible based on attribute set *A* ∈ *At* if they share the same values on all attributes in *A*.

The equivalence relation may be used to create equivalence classes which induces a partition of *U* denoted by *U*/*E*. An equivalence class with an object *x* is given by [*x*] = {*y* ∈ *U*|*xEy*}. The fundamental notion of rough set theory, i.e., approximations and the three regions are defined using equivalence classes as follows.

$$\begin{array}{ccc} \underline{apr}\left(C\right) & = & \{x\in U\;\;|\;\;[x]\subseteq C\}, \end{array}$$ (3)

$$\begin{array}{ccc} \bar{apr}\left(C\right) & = & \{x\in U\;\;|\;\;[x]\cap C\neq ⌀\}. \end{array}$$ (4)

The lower and upper approximations are used to define the positive, negative and boundary regions (which leads to three-way decisions, already discussed in the section **Three-way Decisions**) given by [24, 55],

$$\begin{array}{ccc} \text{POS}(C) & = & \underline{apr}\left(C\right)\\ & = & \{x\in U\;\;|\;\;[x]\subseteq C\}, \end{array}$$ (5)

$$\begin{array}{ccc} \text{NEG}(C) & = & {\left(\bar{apr}\left(C\right)\right)}^{c}\\ & = & \{x\in U\;\;|\;\;[x]\cap C=⌀\}, \end{array}$$ (6)

$$\begin{array}{ccc} \text{BND}(C) & = & \bar{apr}\left(C\right)-\underline{apr}\left(C\right)\\ & = & \{x\in U\;\;|\;\;[x]⊈C,[x]\cap C\neq ⌀\}. \end{array}$$ (7)

The three regions has a simple but very meaningful interpretation. We accept an object as belonging to the concept if it is in the positive region. We reject an object as belonging to the concept if it is in the negative region. We defer the decision for an object as belonging to the concept if it is in the boundary region. The three regions representation of rough sets defined in Eqs (5)–(7) has lead to the introduction of the theory of three-way decisions [39]. In fact, the major notion of three-way decisions, i.e., the division of universal set into three regions is borrowed from rough sets.

The deferment decision option which is exercised based on the boundary region is useful in at least two aspects. Firstly, it provides hints for seeking and incorporating anticipated future information in the decision making model for making decisions on the deferred cases. It is hoped that as information matures, the number of deferred cases will reduce thereby leading to more precise decisions. Secondly, the deferred cases which are typically associated with high levels of uncertainty and therefore, no obvious immediate decisions, the deferment decision option may help avoiding some false decisions. The former aspect is of particular interest from protein functions classification perspective We further elaborate this in the next section.

### Three-way decisions and evolving information

The information describing the functions of proteins are evolving. An interesting issue is how to build effective decision making model for taking advantage of evolving information of protein functions. In this section, we elaborate the role of rough sets based three-way decisions as one of the possibility. In particular, we explain, how evolving information leading to new features can be effectively utilized in three-way decision making based on rough sets. We consider a demonstrative example for this purpose based on an information table of Table 1.

**Table 1:** An information table for proteins.

Objects	Localization available at *t*_0_	Interacting proteins available at *t*_1_	No. of Domains available at *t*_2_	Function
*P*_1_	Mitochondria	0	0	Yes
*P*_2_	Mitochondria	0	1	No
*P*_3_	CytoPlasm	1	0	Yes
*P*_4_	CytoPlasm	2	0	No
*P*_5_	CytoPlasm	2	0	No
*P*_6_	CytoPlasm	0	0	Yes
*P*_7_	CytoPlasm	2	1	No
*P*_8_	Mitochondria	0	1	No

The rows of the Table 1 represent the proteins labeled as *P*_*i*_’s and the columns describe the feature or characteristics of proteins. The last column labeled as “Function” represents the decision attribute. The Function = Yes means that a protein performs the function and Function = No means that the protein does not perform the function. Let us assume three instances in time, i.e., *t*_0_, *t*_1_ and *t*_2_ with *t*_0_ < *t*_1_ < *t*_2_. Further assume that at time instance *t*_0_, we only have information about the “Localization” of the proteins. At time instance *t*_1_, we have additional information of “interacting proteins” of the proteins and at time instance *t*_2_, we have more additional information about the “No. of Domains” of the proteins. Using the information available at time instance *t*_0_, i.e., “Localization”, we have the following equivalence classes.

$$\begin{array}{c} \left\{O_{1},O_{2},O_{8}\right\},\left\{O_{3},O_{4},O_{5},O_{6},O_{7}\right\}, \end{array}$$ (8)

Using the above equivalence classes, we can compute the positive, negative and boundary regions using Eqs (5)–(7). The three regions are given by,

$$\begin{array}{ccc} \text{POS(C)} & = & \emptyset ,\\ \text{NEG(C)} & = & \emptyset ,\\ \text{BND(C)} & = & \{O_{1},O_{2},O_{3},O_{4},O_{5},O_{6},O_{7},O_{8}\}, \end{array}$$ (9)

In the same way we can compute the three regions at time instances *t*_1_ and *t*_2_, when we have additional information in the form of “interacting proteins” and “No. of Domains”. Table 2 summarizes the three regions corresponding to the information available at the three instances of time. Looking at the three regions for the different time instances, we may note that objects in the boundary region are decreasing and are becoming part of the positive or negative regions as more information is available at time instances of *t*_1_ and *t*_2_. In other words, the additional information about the “interacting proteins” and “No. of Domains” of the objects has increased the size of the positive and negative regions. This means that we can make more decisions in the form of acceptance or rejection when the level of available information increases. In this article, we argue that this property of three-way decision making can be quite useful for making decisions on protein functions classification.

**Table 2:** Property of the three regions with evolving information.

	Localization time *t*_0_	Interacting proteins time *t*_1_	No. of Domains time *t*_2_
POS(C)	∅	{*O*_3_, *O*_6_}	{*O*_1_, *O*_3_, *O*_6_}
NEG(C)	∅	{*O*_4_, *O*_5_, *O*_7_}	{*O*_2_, *O*_4_, *O*_5_, *O*_7_, *O*_8_}
BND(C)	{*O*_1_, *O*_2_, …, *O*_8_}	{*O*_1_, *O*_2_, *O*_8_}	∅

**Fig 3:**
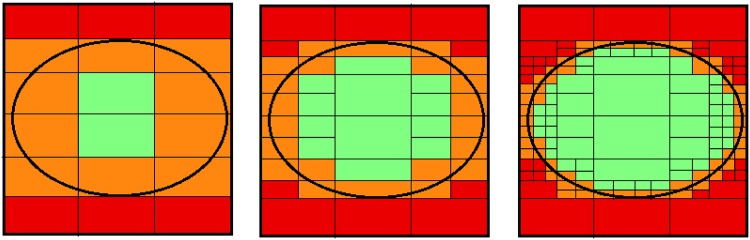
The three regions with evolving information. The sub-figures from left to right should be read as a, b and c respectively.

In order to see the same phenomena visually, we include Fig 3. In each sub figure, the green, red and orange colours represents the positive, negative and boundary regions. The circle represents a certain concept and the small rectangles depict the equivalence classes. From Fig 3(a), we have least information and in Fig 3(c), we have most information. In Fig 3(b), we have moderate level information. We may note that as information matures, we have finer level details leading to refined partitions. This is shown by the smaller sized boxes in Fig 3(b) and 3(c). The finer level details due to additional information enables us to move some of the equivalence classes from boundary to either positive or negative regions thereby increasing their respective sizes. This leads to fine tuning of positive and negative regions and we gradually converge to the concept, i.e., the circle (Fig 3(c)).

### Three-way decisions using probabilistic rough sets

The conventional rough set model (outlined in Section **Three-way Decisions using Rough Sets**) provide a useful approach for inducing and making three-way decisions. However, this conventional rough sets model which is also sometimes referred to as Pawlak rough set model suffers from a key limitation. Researchers argue that the conditions in the upper and lower approximations may be too strict when it comes to real applications. Specifically the conditions in Eqs (3) and (4), i.e., [*x*] ⊆ *C* and [*x*] ∩ *C* ≠ ⌀ reflecting whether [*x*] is fully contained in *C* and whether [*x*] has some overlap with *C*, respectively, may be too restricted in the sense that they ignore the degree of an overlap between a set and a concept. To overcome this difficulty, many researchers proposed different extensions of rough sets. The probabilistic rough sets represent one class of such extensions and include decision-theoretic rough sets, variable precision rough sets, 0.5-probabilistic rough sets, Bayesian rough sets, information-theoretic rough sets and game-theoretic rough sets.

The general form of probabilistic rough sets resulted from the studies on decision-theoretic rough sets [56, 57]. The probabilistic lower and upper approximations for a concept *C* are defined using a pair of thresholds (*α*, *β*) as [58],

$$\begin{array}{ccc} {\underline{apr}}_{\left(\alpha ,\beta \right)}\left(C\right) & = & \{x\in U\;|\;P(C|[x])\geq \alpha \}, \end{array}$$ (10)

$$\begin{array}{ccc} {\bar{apr}}_{\left(\alpha ,\beta \right)}\left(C\right) & = & \{x\in U\;|\;P(C|[x])>\beta \}, \end{array}$$ (11)

where *P*(*C*|[*x*]) denotes the conditional probability of a concept *C* with an equivalence class [*x*]. Given that an object *x* ∈ [*x*], the conditional probability highlights the evaluation of an object *x* to be in *C*. The three rough set regions based on lower and upper approximations are defined as,

$$\begin{array}{ccc} {\text{POS}}_{\left(\alpha ,\beta \right)}\left(C\right) & = & \{x\in U|P(C|[x])\geq \alpha \}, \end{array}$$ (12)

$$\begin{array}{ccc} {\text{NEG}}_{\left(\alpha ,\beta \right)}\left(C\right) & = & \{x\in U|P(C|[x])\leq \beta \}, \end{array}$$ (13)

$$\begin{array}{ccc} {\text{BND}}_{\left(\alpha ,\beta \right)}\left(C\right) & = & \{x\in U|\beta <P(C|[x])<\alpha \}. \end{array}$$ (14)

The POS_(*α*,*β*)_(*C*), NEG_(*α*,*β*)_(*C*) and BND_(*α*,*β*)_(*C*) in Eqs (12)–(14) are referred to as positive, negative and boundary regions, respectively. Based on how these thresholds are determined and interpreted we have different probabilistic rough set models.

To demonstrate the use of three-way decisions for proteins functions classification, we focus on two probabilistic rough set models, namely, GTRS [25–27] and ITRS [59]. These two models have at least two advantages over other models.

- Firstly, compared to some of the earlier probabilistic models, such as, 0.5-probabilistic rough set model and (0.5, *β*) model, where due to restricted pairs of thresholds, the determination and interpretation of thresholds are ignored, the GTRS and ITRS allows for investigation and examination of thresholds based on different aspects.
- Secondly, unlike other models that require user intervention to set the thresholds, such as, decision-theoretic rough sets and variable precision rough sets, the GTRS and ITRS can be used to learn and set the thresholds automatically when combined with some typical search mechanism [25].

For the sake of being complete, we briefly explain and discuss the GTRS and ITRS models.

#### Three-way decisions using game-theoretic rough sets

The game-theoretic rough sets or GTRS utilizes game-theoretic formulation to determine thresholds of probabilistic rough sets [26, 27]. In particular, the thresholds are interpreted based on a tradeooff solution between multiple criteria employed in a game setting for analyzing rough sets [25–27]. A typical game in GTRS has three essential components, i.e., game players, strategies and payoff or utility functions. These components are generally represented as a tuple {*P*, *S*, *u*}. We now explain each of them.

**Game players:** The players in the game are denoted by a set *P*. Generally, there can be *n* players in a game. However, for the sake of simplicity, a two player game is commonly considered in GTRS. Based on the overall game objective and goals, we may have different types of game players. For instance, in a previous game for analyzing region uncertainty, the players were defined as the uncertainty of the immediate and deferred decision regions and in another game that seek for a balanced rough set model, the players of accuracy and generality were used [25, 60]. In general, the players in the game are selected to reflect the overall purpose of the game. In GTRS, the players are defined as different aspects and properties of rough sets based classification and decision making such as accuracy, generality, precision and uncertainty.

**Strategies:** Each player in the game participate by playing different strategies. The set of strategies available to player *i* is denoted by *S*_*i*_. The Cartesian product of all possible strategy sets is denoted by *S* = *S*_1_ × *S*_2_ × … × *S*_*n*_, where *S* contains ordered pairs of the form (*s*_1_, *s*_2_, …, *s*_*n*_) such that *s*_1_ ∈ *S*_1_, *s*_2_ ∈ *S*_2_ and *s*_*n*_ ∈ *S*_*n*_. Each order pair in *S* is called a strategy profile and represents a certain situation encountered in a game.

The strategies in GTRS are realized as different changes and modifications in the (*α*, *β*) thresholds. Depending on the initial values of thresholds, we may have different types of strategies. For instance, if the initial values of (*α*, *β*) are set to (1, 0.5), then the strategies may be formulated as decreasing levels of *α* and *β*. Alternatively, when the initial values of (*α*, *β*) are set to (1, 0), then the strategies may be formulated as decreasing levels of *α* and increasing levels of *β*. Please note that in order to keep the regions disjoint, it is assumed that 0 ≤ *β* < *α* ≤ 1.0. The strategies of the players in a game lead to effective modification of the thresholds which ultimately determines the final configuration of the thresholds.

**Payoff functions:** The payoff functions for the players are represented by a set *u* = (*u*_1_, …, *u*_*n*_). Each *u*_*i*_ is a real valued utility function for player *i* and it maps the strategy profiles to real values, i.e., *u*_*i*_: *S* ↦ ℜ. In particular, the payoffs reflect the utilities of performing or selecting a certain strategy. Recall the game players in GTRS which are represent different aspects or properties of rough sets, the payoff function for a certain player is based on particular measure employed for evaluating its respective property.

In a game setting, every player wants to perform a strategy that will maximize its payoff. The selected strategies of the players however affect their opponents payoffs. The game solution is used to choose a balanced and trade off point based on the utilities of all the players. The game solution of Nash equilibrium is commonly used in GTRS for this purpose.

Considering a strategy profile *s*_−*i*_ = (*s*_1_, *s*_2_, …, *s*_*i*−1_, *s*_*i*+1_, …, *s*_*n*_), which means a strategy profile without player *i* strategy. Moreover, the strategy profile (*s*_1_, *s*_2_, …, *s*_*n*_) may be denoted in revised notation as (*s*_*i*_, *s*_−*i*_). The strategy profile (*s*_1_, *s*_2_, …, *s*_*n*_) = (*s*_*i*_, *s*_−*i*_) is a Nash equilibrium if [61],

$$\begin{array}{c} \forall i,\forall s_{i}^{{}^{\prime }}\in S_{i},\;\;\;u_{i}\left(s_{i},s_{-i}\right)\geq u_{i}\left(s_{i}^{{}^{\prime }},s_{-i}\right),\;\;\;\;\text{where}\left(s_{i}^{{}^{\prime }}\neq s_{i}\right) \end{array}$$ (15)

This means that for all players *i*, their respective strategies, i.e., *s*_*i*_ is the best response to *s*_−*i*_. In other words, a strategy profile constitutes a Nash equilibrium when no player is benefited from changing his strategy alone.

The above game description is used in GTRS to formulate a game. However, with a single one time and non-repeated game, we may not be able to reach effective thresholds that fulfill the demands of the underlying applications. We need to repeat the game. The essential idea is to repeatedly modify and refine the thresholds, until we achieve certain performance criteria. By formulating a game and utilizing the notions such as game solution and repetitive games, the GTRS seek for an effective configuration of the threshold levels that are employed in the probabilistic rough sets framework to induce three-way decisions.

#### Three-way decisions using information-theoretic rough sets

The Information-theoretic rough sets (or ITRS) approach the threshold determination issue from the viewpoint of minimizing the information uncertainty of the probabilistic rough set regions [59]. Let Δ_*P*_(*α*, *β*), Δ_*N*_(*α*, *β*) and Δ_*B*_(*α*, *β*) denote the overall uncertainties of the probabilistic positive, negative and boundary regions respectively. The ITRS is based on minimization or optimization of the following problem.

$$\begin{array}{ccc} & \text{arg}\min_{\left(\alpha ,\beta \right)}\Delta \left(\alpha ,\beta \right),\;\;\;\;\;\;\text{where,} & \\ & \Delta \left(\alpha ,\beta \right)=\Delta_{P}\left(\alpha ,\beta \right)+\Delta_{N}\left(\alpha ,\beta \right)+\Delta_{B}\left(\alpha ,\beta \right) \end{array}$$ (16)

Please be noted that we used slightly modified notations that were reported in [25]. Eq (16) suggests that we seek thresholds (*α*, *β*) that will minimize the uncertainty of the three regions.

The overall uncertainty in Eq (16) is typically considered as an average uncertainty of the three regions [59].

$$\begin{array}{cc} & \Delta_{P}\left(\alpha ,\beta \right)=P\left({\text{POS}}_{\left(\alpha ,\beta \right)}\left(C\right)\right)\delta_{P}\left(\alpha ,\beta \right), \end{array}$$ (17)

$$\begin{array}{cc} & \Delta_{N}\left(\alpha ,\beta \right)=P\left({\text{NEG}}_{\left(\alpha ,\beta \right)}\left(C\right)\right)\delta_{N}\left(\alpha ,\beta \right), \end{array}$$ (18)

$$\begin{array}{cc} & \Delta_{B}\left(\alpha ,\beta \right)=P\left({\text{BND}}_{\left(\alpha ,\beta \right)}\left(C\right)\right)\delta_{B}\left(\alpha ,\beta \right), \end{array}$$ (19)

where *δ*_*P*_(*α*, *β*), *δ*_*N*_(*α*, *β*) and *δ*_*B*_(*α*, *β*) are the uncertainties of the three regions which may be computed and interpreted using different measures of uncertainties. Moreover, *P*(POS_(*α*,*β*)_(*C*)), (POS_(*α*,*β*)_(*C*)) and *P*(POS_(*α*,*β*)_(*C*)) are the probabilities of the three regions. Two measures, i.e., Shannon entropy and gini coefficient are being previously employed for interpreting and measuring the uncertainties of the three regions, i.e., *δ*_*P*_(*α*, *β*), *δ*_*N*_(*α*, *β*) and *δ*_*B*_(*α*, *β*). We now define each of them.

Consider a partition based on a concept *C*, given by, *π*_*C*_ = {*C*, *C*^*c*^} and another partition with respect to the thresholds (*α*, *β*), given by, *π*_(*α*,*β*)_ = {POS_(*α*,*β*)_(*C*), NEG_(*α*,*β*)_(*C*), BND_(*α*,*β*)_(*C*)}. The uncertainty in *π*_*C*_ with respect to the three probabilistic regions based on Shannon entropy is given by, [59],

$$\begin{array}{ccc} \delta_{P}\left(\alpha ,\beta \right) & = & H\left(\pi_{C}|{\text{POS}}_{\left(\alpha ,\beta \right)}\left(C\right)\right)=-P\left(C|{\text{POS}}_{\left(\alpha ,\beta \right)}\left(C\right)\right)\;\text{log}\;P\left(C|{\text{POS}}_{\left(\alpha ,\beta \right)}\left(C\right)\right)\\ & & -P\left(C^{c}|{\text{POS}}_{\left(\alpha ,\beta \right)}\left(C\right)\right)\;\text{log}\;P\left(C^{c}|{\text{POS}}_{\left(\alpha ,\beta \right)}\left(C\right)\right), \end{array}$$ (20)

$$\begin{array}{ccc} \delta_{P}\left(\alpha ,\beta \right) & = & H\left(\pi_{C}|{\text{NEG}}_{\left(\alpha ,\beta \right)}\left(C\right)\right)=-P\left(C|{\text{NEG}}_{\left(\alpha ,\beta \right)}\left(C\right)\right)\;\text{log}\;P\left(C|{\text{NEG}}_{\left(\alpha ,\beta \right)}\left(C\right)\right)\\ & & -P\left(C^{c}|{\text{NEG}}_{\left(\alpha ,\beta \right)}\left(C\right)\right)\;\text{log}\;P\left(C^{c}|{\text{NEG}}_{\left(\alpha ,\beta \right)}\left(C\right)\right), \end{array}$$ (21)

$$\begin{array}{ccc} \delta_{P}\left(\alpha ,\beta \right) & = & H\left(\pi_{C}|{\text{BND}}_{\left(\alpha ,\beta \right)}\left(C\right)\right)=-P\left(C|{\text{BND}}_{\left(\alpha ,\beta \right)}\left(C\right)\right)\;\text{log}\;P\left(C|{\text{BND}}_{\left(\alpha ,\beta \right)}\left(C\right)\right)\\ & & -P\left(C^{c}|{\text{BND}}_{\left(\alpha ,\beta \right)}\left(C\right)\right)\;\text{log}\;P\left(C^{c}|{\text{BND}}_{\left(\alpha ,\beta \right)}\left(C\right)\right). \end{array}$$ (22)

Where we used the additional notations *H*(*π*_*C*_|POS_(*α*,*β*)_(*C*)), *H*(*π*_*C*_|POS_(*α*,*β*)_(*C*)) and *H*(*π*_*C*_|POS_(*α*,*β*)_(*C*)) to be consistent with the earlier notations [59]. The measure of gini coefficient is also used in the same way to determine the uncertainties of the three regions [62]. The uncertainties of the three regions are computed as [62],

$$\begin{array}{ccc} \delta_{P}\left(\alpha ,\beta \right) & = & G\left(\pi_{C}|{\text{POS}}_{\left(\alpha ,\beta \right)}\left(C\right)\right)=1-P{\left(C\right|{\text{POS}}_{\left(\alpha ,\beta \right)}\left(C\right))}^{2}\\ & & -P{\left(C^{c}|{\text{POS}}_{\left(\alpha ,\beta \right)}\left(C\right)\right)}^{2}, \end{array}$$ (23)

$$\begin{array}{ccc} \delta_{N}\left(\alpha ,\beta \right) & = & G\left(\pi_{C}|{\text{NEG}}_{\left(\alpha ,\beta \right)}\left(C\right)\right)=1-P{\left(C|{\text{NEG}}_{\left(\alpha ,\beta \right)}\left(C\right)\right)}^{2}\\ & & -P{\left(C^{c}|{\text{NEG}}_{\left(\alpha ,\beta \right)}\left(C\right)\right)}^{2}, \end{array}$$ (24)

$$\begin{array}{ccc} \delta_{B}\left(\alpha ,\beta \right) & = & G\left(\pi_{C}|{\text{BND}}_{\left(\alpha ,\beta \right)}\left(C\right)\right)=1-P{\left(C|{\text{BND}}_{\left(\alpha ,\beta \right)}\left(C\right)\right)}^{2}\\ & & -P{\left(C^{c}|{\text{BND}}_{\left(\alpha ,\beta \right)}\left(C\right)\right)}^{2}. \end{array}$$ (25)

Please note again that the notation *G*(*π*_*C*_|BND_(*α*,*β*)_(*C*)), *G*(*π*_*C*_|BND_(*α*,*β*)_(*C*)) and *G*(*π*_*C*_|BND_(*α*,*β*)_(*C*)) are being used for the sake of being consistent with the previous notations [62].

The ITRS is generally combined with a searching mechanism to determine effective thresholds. In particular, the minimization of overall uncertainty in Eq (16), is used to guide the search towards optimal thresholds. Recently, the gradient descent approach was suggested in this regards [59].

### Three-way decision algorithm for classifying protein

In this section, we look at three-way decision approach from implementation perspective. Algorithm 1 is presented for this purpose. The algorithm explains how three-way decisions can be used in classifying proteins with evolving information.

**Algorithm 1** Iterative Three-way decision making algorithm

**Input:** An information table containing a new feature and POS_(*α*,*β*)_(*C*), NEG_(*α*,*β*)_(*C*), and BND_(*α*,*β*)_(*C*) based on information from previous features

**Output:** Updated regions, POS_(*α*,*β*)_(*C*), NEG_(*α*,*β*)_(*C*) and BND_(*α*,*β*)_(*C*)

1: **if** *Q*_*P*_(*α*, *β*) ≥ *c*_1_ and *Q*_*N*_(*α*, *β*) ≥ *c*_2_ **then**

2:  Determine thresholds (*α*′, *β*′) using GTRS and ITRS for information table with *U* = BND_(*α*,*β*)_(*C*)

3:  POS_(*α*′, *β*′)_(*C*) = {*x* ∈ BND_(*α*,*β*)_(*C*)|*P*(*C*|[*x*]) ≥ *α*′}

4:  ${\text{NEG}}_{\left(\alpha_{,}^{\prime }\beta^{\prime }\right)}\left(C\right)=\left\{x\in {\text{BND}}_{\left(\alpha ,\beta \right)}\left(C\right)|P\left(C|\left[x\right]\right)\leq \beta^{\prime }\right\}$

5:  BND_(*α*′, *β*′)_(*C*) = {*x* ∈ BND_(*α*,*β*)_(*C*)|*β*′ < *P*(*C*|[*x*]) < *α*′}

6:  POS_(*α*,*β*)_(*C*) = POS_(*α*′, *β*′)_(*C*)⋃POS_(*α*,*β*)_(*C*)

7:  NEG_(*α*,*β*)_(*C*) = NEG_(*α*′, *β*′)_(*C*)⋃NEG_(*α*,*β*)_(*C*)

8:  BND_(*α*,*β*)_(*C*) = BND_(*α*′, *β*′)_(*C*)−BND_(*α*,*β*)_(*C*)

9: **else**

10:  Determine thresholds (*α*, *β*) using GTRS and ITRS.

11:  POS_(*α*,*β*)_(*C*) = {*x* ∈ *U*|*P*(*C*|[*x*]) ≥ *α*}

12:  NEG_(*α*,*β*)_(*C*) = {*x* ∈ *U*|*P*(*C*|[*x*]) ≤ *β*}

13:  BND_(*α*,*β*)_(*C*) = {*x* ∈ *U*|*β* < *P*(*C*|[*x*]) < *α*}

14: **end if**

15: **return** POS_(*α*,*β*)_(*C*), NEG_(*α*,*β*)_(*C*), BND_(*α*,*β*)_(*C*)

The algorithm accepts information table containing information about a new feature and the three regions based on the previous features, i.e., positive, negative and boundary regions denoted as POS_(*α*,*β*)_(*C*), NEG_(*α*,*β*)_(*C*), and BND_(*α*,*β*)_(*C*), respectively. In line 1, the algorithm evaluates the positive and negative regions by employing some quality criteria denoted as *Q*_*POS*_(*α*,*β*) and *Q*_*NEG*_(*α*,*β*) (representing some quality related aspect of the positive and negative regions, respectively). These notations are introduced to represent the general notion of any criteria that is employed for evaluating the three regions. They may be interpreted in terms of cost, risks, uncertainty, accuracy or precision. The quality of the regions may be measured based on the notions such as risks, cost, uncertainty, accuracy or precisions. As discussed in the previous subsection titled **Three-way Decisions and Evolving Information**, when the features evolve, the positive region gradually converges to the concept *C* (i.e., more precisely reflect the region representing the concept) and the negative region gradually converges to the complement of the concept *C*^*c*^ (i.e., more precisely reflect the region not in the concept), respectively. As a result, the quality of the two regions improves. As improvement in quality is a gradual process in this case, at the current level of information, the quality of the positive and negative regions may or may not be effective (please be noted that the term effective here may have different interpretation based on the underlying applications). We deal with these two cases separately.

**Fig 4:**
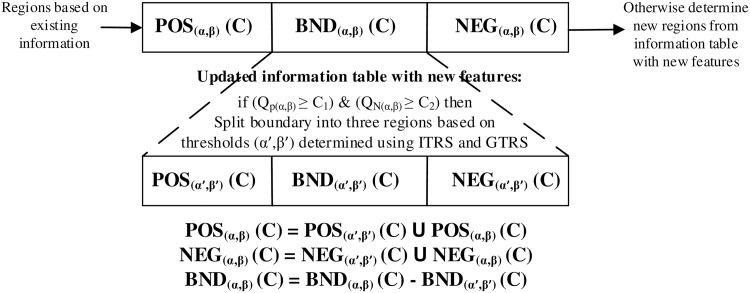
Visualization of iterative three-way decision making algorithm.

If the quality of the regions are above some acceptable levels *c*_1_ and *c*_2_, we will only examine the objects in the boundary region and will not further investigate the positive and negative regions. The boundary is expected to shrink further as we have access to new features. In any other case, we will examine the full information table to obtain the three regions. In other words, we are not satisfied with the quality of the positive and negative regions (they are below the levels *c*_1_ and *c*_2_) and we expect that additional information may improve their respective quality levels. We first deal with the former case. In line 3, we determine thresholds based on the reduced information table with *U* = BND_(*α*,*β*)_(*C*). As new information becomes available in the form of a new feature, we may be able to confidently classify further objects in the boundary. This is shown in line 4-7 where we further divide the objects in the boundary region. In line 6-8, we update the three regions based on further examination of the boundary. From line 10-13 we examine the case when the positive and negative regions based on the previous knowledge were not of acceptable quality. We therefore examine the full information table and update the three regions accordingly. The Fig 4 represents the essential ideas of the algorithm 1 in diagrammatic form.

It may be noted that the constants *c*_1_ and *c*_2_ may be defined in different ways depending on the application needs and requirements. For instance, if we want to reduce the processing overload, we may define them moderately. On the other hand, if processing overload is not an issue and we are more concerned about the accuracy, then we may define them more strictly. Other ways in which they may be defined are by making comparison with the quality of the regions obtained with the standard Pawlak models or other known models in the domains or by considering the improvement in quality based on the new features.

## Experiment setup

### Data preparation

To evaluate the use of three-way approach, we examine the application of three-way decisions on well studied *Saccharomyces cerevisiae* species proteins [63, 64], obtained from most widely used *Uniport* database [29]. From various classification schemes developed to standardize the descriptions of protein functions, we chose the state of the art Gene Ontology (GO) [30] classification scheme. The gene ontology is a structured, controlled vocabulary of protein functions also called terms. GO terms provide consistency in annotating protein roles in the cellular context. It is arranged in a DAG (please refer to Section Background) structure in which each node of the graph represents a unique functional term and each term is arranged in a parent child relationship with other terms. The child term either *is a* special case of the parent or is a *part of* the parent process i.e., a sub-process or component. For the evaluation of our methodology we operate on *molecular function* category of gene ontology. To reveal the evolving nature of biological information, we present features in the order in which they are evolved over the time i.e., most basic type of information is presented first and so on [3]. For classifying a protein into one or more molecular function terms of gene ontology, we retrieve ten different types of features from varied biological databases. Each feature is helpful in characterizing one or more functional categories and is represented by the symbol *Fi*.

**Protein Sequence Length (*F*_1_)**: In every cell, genes are converted into proteins via the processes of transcription and translation also called the central dogma of molecular biology. The end product of these processes is a sequence built from twenty amino acids, and is commonly known as the primary structure of a protein. The amino acid sequence is the most basic type of information available about a protein, as it can provide concrete evidence about different characteristics of a protein such as its binding sites, sub-cellular localization, structure and function. To quantify these biological aspects of a protein, we use feature (namely *F*_1_), as the length of protein sequence which is extracted from *Uniprot* database [29].

**Protein Localizations (*F*_2_)**: The location of a protein in the cell can also be associated with its function. Co-localized proteins are more likely to be part of same molecular activity. Likewise, proteins localizing in many different locations can be part of diverse activities. To capture this aspect, we calculate feature *F*_2_ as the number of locations a protein can localize. The protein localization data is retrieved from the *Uniprot* database [29].

**Biological Processes (*F*_3_)**: A biological process refers to the series of events performed by one or more assemblies of molecular functions with a defined beginning and end. A protein participating in many biological processes is more likely to have many molecular level roles. Thus the number of biological processes of a protein can also be used to capture the molecular level activities of a protein. As a third feature (*F*_3_), we count the number of biological processes of a protein. It is obtained by retrieving counts of Biological Process ontology terms from the Gene Ontology database [30].

**Number of Interacting Proteins (*F*_4_)**: For calculating the fourth feature of our method, namely *F4*, we use genome wide protein-protein interactions (PPI) data to predict proteins function. In a living cell, protein-protein interactions are amongst the most ubiquitous types of interactions and their precise knowledge helps in understanding the activities performed by a protein as well as the processes it is part of. A protein having many different interacting partners can be said to be part of many different functions. Thus the number of interactions (*F*_4_) of a protein can be linked to the wide variety of activities it performs. We obtain PPI data from most widely used PPI databases, namely, *IntAct* [65] and *STRING* [66]. Since protein-protein interactions databases are noisy, we only consider interactions that are experimentally verified and are supported by at least two experiments.

**Number of Domains (*F*_5_)**: Protein domains are the sequential units that fold in a particular shape, making independent structures in different proteins. Several classification schemes have been proposed e.g., [67] to define and demarcate different domains of which some based on clustering conserved subsequences into related domain families, others on known distinct structural classes [68]. One of the most famous and widely used domain classification schema is the *Interpro* database [69]. *InterPro* database contains diagnostic signatures of protein sequences consisting of models e.g., regular expressions models, Hidden Markov Models etc., which describe protein domains found within sequence. Domains are the most important feature among relevant sequence features of a protein that associate it to a particular kind of functions. To integrate domain relevance we also use as a feature (namely *F*_5_) the number of *Interpro* conserved domains within a query protein sequence.

**Number of Conserved Motifs (*F*_6_)**: A motif is a conserved amino acid sequence pattern in a protein sequence that may be associated to a specific function. These subsequences may often contain small “gaps” of fixed or variable lengths among amino acids of the subsequence. The knowledge of exact patterns of motifs and their functions is helpful in the understanding of structure and function of related proteins in which such motifs may appear. For example, if a motif of a certain family is present in a protein sequence then it will make it highly probable to functionally associate that protein with the functions of that motif i.e., we can associate proteins with functions by merely checking the presence of certain motifs. Thus in our technique, as sixth feature (*F*_6_) we count the number of conserved motifs in a protein sequence using *Prosite* motif database [70].

**Number of Protein Structures (*F*_7_)**: A protein’s primary structure consists of sequence of amino acids. These amino acids due to their varied physical and chemical properties as well as the presence of different participant cellular forces, assumes a unique configuration in three-dimensional space. This stable configuration of proteins is also called the tertiary structure of proteins. This final configuration or structure of a protein is strongly correlated to its function, because in many biological processes, the interacting proteins have to come into physical contact in order to accomplish the desired function. The structure of a protein also determines many of its functional characteristics, for example its inter-facial binding sites, the specific ligands it binds to, cellular localizations, as well as other proteins it can interact with. Among all the structural databases PDB (Protein Data Bank) [71] is by far the most reliable, wide-ranging as well as popular repository for experimentally derived protein 3D structures. We query the PDB database to obtain the number of experimentally determined structures associated to a protein under investigation and use this information as a feature (namely *F*_7_) to characterize its function.

**Molecular Weight of Protein (*F*_8_)**: Although weight of a protein is not strongly related to its function but in some cases it can be used to generally group them into broader functional categories. We retrieve Molecular weight rounded to the nearest mass unit (Dalton) from Uniprot Database [29] and use it as a feature (namely *F*_8_) for our 3-way classifier.

**Number of Interfacing Residues in Protein Structure (*F*_9_)**: Many proteins bind together and form multi-protein complexes. Different proteins in the complex perform different functions. These functions are associated with the number of residues on a protein’s interface that enables it to stabilize, bind and form complexes. Owing to the significance of interfacing residues we utilize a structural feature i.e., the number of residues on the protein’s interface to characterize function of a protein. The interfacing residues can vary for various functional activities. To capture this aspect we used *PDBe PISA* server [72], to retrieve the number of predicted interfacing residues and use it as feature (namely *F*_9_) for our 3-way classifier.

**Binding sites in the Predicted Interface (*F*_10_)**: A protein’s physical interaction with other molecules, determines its biological activities. For example antibody proteins selectively bind to viruses or bacteria to choose them for destruction, the hexokinase protein binds to ATP molecule as well as with glucose molecule in order to catalyze their chemical reaction, and so on. Without any doubt almost all proteins stick, or bind, to other molecules in order to perform their activities at molecular level. Some proteins bind very tightly while others bind for a short period of time depending on their specificity as well as the molecular task they have to perform. Each protein can usually bind to one or few other molecules determined by the nature of binding residues (also called binding sites) at its surface. To determine the specificity of a protein for binding and performing wide variety of functions we calculate a feature (namely (*F*_10_), which is the number of binding sites on its surface that are predicted using *PDBeFold* Server [73].

The above features namely, *F*_1_ to *F*_10_, are extracted using the Feature Extractor module (already described in Section **An Architecture of Protein Function Classification with Three-way Decisions**), from the world wide biological databases using the knowledge discovery module. The Feature Extractor module also has the capability to incorporate any new feature, say *F*_11_ in the predictive task. To imitate the ever evolving nature of biological information, we selected and ranked features from most basic type to the latest type i.e, *F*_1_ namely, sequence similarity, is a basic type of feature and *F*_10_ namely, number of binding sites on a protein interface, is a specific feature known after information evolution.

### Three-way approaches used in the experiments

We performed experiments with five three-way decision making approaches based on GTRS and ITRS. Specifically three of these approaches are based on GTRS and two of them are based on ITRS.

The three approaches with GTRS are based on different games that are formulated based on description in Section **Three-way Decisions using GTRS**. The essential difference in these games are the consideration of different types of game players. Two of these games are based on examining a balance between the uncertainties of probabilistic rough set regions. These games are based on two players, namely, immediate decision region, denoted as *I* and deferred decision region, denoted as *D*. The player *I* reflects the collective uncertainty in probabilistic positive and negative regions and the player *D* denotes the uncertainty in the probabilistic boundary region. By realizing changes in thresholds as game strategies, the players in a game compete in a game by selecting appropriate changes in the thresholds which are used in determining the final settings of the thresholds. Two games are constructed with these game players, i.e., player *I* and *D* by realizing different interpretation and computation of uncertainty. In one game, the uncertainty is measured with the Shannon entropy and in another game it is measured with gini coefficient. These two games will be referred to as GTRS_*E*_ and GTRS_*G*_, respectively. These game were previously examined in the context of text categorization and medical decision making [25, 62, 74]. The third game in GTRS is based on determining a trade off between two aspects of rough sets based classification, namely, accuracy and generality. This game was previously examined in the context of recommender systems in [60]. We will refer to this game as GTRS_(*A*,*G*)_.

Two approaches are considered with the ITRS. These two approaches are ITRS based on Shannon entropy and ITRS based on Gini coefficient as discussed in Section **Three-way Decisions using ITRS**. We denote these approaches as ITRS_*E*_ and ITRS_*G*_, respectively. Both of these measures interpret the uncertainty in a different way and therefore will lead to different thresholds.

In all experiments, we considered the top five most frequent protein functions in the database. For each protein function (recall that each protein function is considered as a category), we learn the probabilistic thresholds (*α*, *β*) and performed three-way decisions using the five approaches discussed above in this section. We considered four feature sets. In each feature set, we consider the features whose relevant information was previously available or which emerged roughly at the same time. In particular, the first feature set comprise of *F*_1_, *F*_2_ and *F*_3_ (please refer to Section **Data Preparation** for their details). We denote the first feature set as *FS*_1_. The second feature set denoted as *FS*_2_, is given by *FS*_1_ ∪ *F*_4_. The third and fourth feature sets, denoted as *FS*_3_ and *FS*_4_ are given by *FS*_3_ = *FS*_2_ ∪ {*F*_5_, *F*_6_} and *FS*_4_ = *FS*_3_ ∪ {*F*_7_, *F*_8_, *F*_9_, *F*_10_}, respectively. Please be noted that the *FS*_1_ contains the oldest available information about proteins while *FS*_4_ is the represents the most recent information comprising the previous knowledge and newly evolved information. Finally, all the results are based on 10 folds cross validation.

## Results and discussion

### Experimental results

We report the results of accuracy and generality for the considered three-way approaches. The accuracy and generality may be defined as [24],

$$\begin{array}{ccc} Accuracy(\alpha ,\beta ) & = & \frac{|\left({\text{POS}}_{\left(\alpha ,\beta \right)}\left(C\right)\cap C\right)⋃\left({\text{NEG}}_{\left(\alpha ,\beta \right)}\left(C\right)\cap C^{c}\right)|}{\left|\text{POS}{}_{\left(\alpha ,\beta \right)}\left(C\right)⋃\;\text{NEG}{}_{\left(\alpha ,\beta \right)}\left(C\right)\right|}, \end{array}$$ (26)

$$\begin{array}{ccc} Generality(\alpha ,\beta ) & = & \frac{|\;{\text{POS}}_{\left(\alpha ,\beta \right)}\left(C\right)⋃{\text{NEG}}_{\left(\alpha ,\beta \right)}\left(C\right)|}{\left|U\right|}, \end{array}$$ (27)

The accuracy highlights the relative number of correct classification decisions for the objects in the universal set and the generality reflects the relative number of objects for whom classification decisions can be made. Table 3 shows the results obtained with the GTRS based approaches. The rows of the table correspond to the results obtained with a particular set of features and the columns correspond to results of accuracy and generality for different approaches. The best results for accuracy and generality against each approach is presented in bold. We may note that the best results for the generality for the three approaches are against the highest feature set size. Moreover, the generality of the three approaches improve as the feature set size is increased. In particular, the generality of GTRS_(*A*,*G*)_ with lowest feature size is 23.77% and the highest feature set size is 68.75%. This represents a total increase of 44.98% in generality. For the other two approaches, i.e., GTRS_*E*_ and GTRS_*G*_, similar increases in generality with values 45.75% and 39.78% are noted based on the lowest feature set size and highest feature set size. Since the features represent the available level of information for predicting protein functions. We may conclude from these results that as the level of information improves (i.e., as we include more features), we are able to make classification decisions for more proteins.

**Table 3:** Results of accuracy and generality for GTRS.

Features	GTRS_(*A*,*G*)_		GTRS_*E*_		GTRS_*G*_	
	Accuracy	Generality	Accuracy	Generality	Accuracy	Generality
*FS*_1_	0.8031	0.2377	**0.7938**	0.2276	0.7969	0.2913
*FS*_2_	**0.8058**	0.3108	0.8077	0.2888	**0.8071**	0.3467
*FS*_3_	0.7808	0.6654	0.7853	0.6737	0.7807	0.6724
*FS*_4_	0.7815	**0.6875**	0.7797	**0.6851**	0.7807	**0.6891**

Let us now look at the results of accuracy in Table 3. We may observe that in general, the values of accuracy decrease slightly as we move from lower to higher feature set sizes. However, compared to the generality, we do not have significant different between these values. For the three approaches, i.e., GTRS_(*A*,*G*)_, GTRS_*E*_ and GTRS_*G*_, the differences between the values of accuracy for the lowest and highest feature set sizes are 2.16%, 1.41% and 1.62%, respectively. From the results of accuracy and generality, we may notice that by increasing the number of features or the level of information, we are able to make more decisions while mainlining the same or similar level of accuracy.

Table 4 shows the results obtained with the ITRS based approaches. The increase in generality for the two approaches, i.e., ITRS_*E*_ and ITRS_*G*_ between the lowest feature set size and highest feature set sizes are 14.36% and 18.29%, respectively. The accuracy values for the ITRS_*E*_ and ITRS_*G*_ approaches are decreased by a small 3.37% and 2.74%, respectively as we increase the feature set size. comparing these results with the GTRS based approaches, we may note that the generality values of the ITRS approaches are significantly better than those obtained with the GTRS based approaches. However, for accuracy there is no significant different between ITRS and GTRS based approaches as both of them are around 80%. Despite some differences in the results with the two approaches, the key observation noted earlier in the discussion of GTRS based results holds for the results in the case of ITRS as well. We may notice again that increasing the number of features or the level of information lead to better generality (which implies more classification decisions) while maintaining the same or similar level of accuracy. In order to highlight this observation, we constructed two figures.

**Table 4:** Results of accuracy and generality for ITRS.

Features	ITRS_*E*_		ITRS_*G*_	
	Accuracy	Generality	Accuracy	Generality
*FS*_1_	**0.8247**	0.6008	**0.8139**	0.5972
*FS*_2_	0.8043	0.6296	0.8101	0.631
*FS*_3_	0.7927	0.7394	0.7878	0.7411
*FS*_4_	0.791	**0.7444**	0.7865	**0.7801**

**Fig 5:**
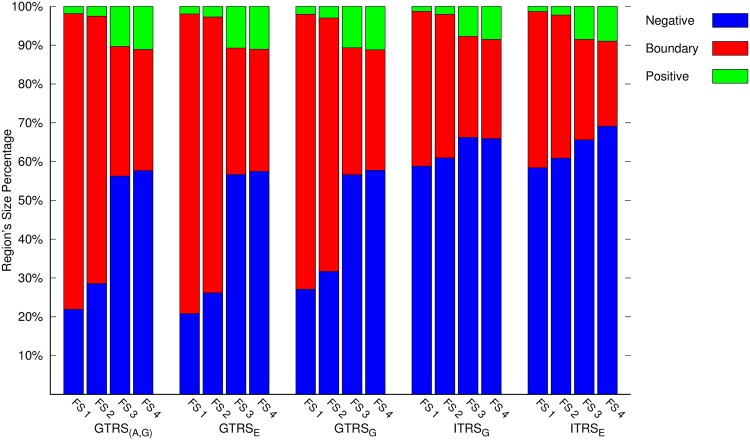
Results of the positive, negative and boundary regions.

Fig 5 shows the results of the positive, negative and boundary regions based on the GTRS and ITRS based approaches. Each bar in the figure is split into three parts, representing the positive, negative and boundary regions respectively. Each set of four bars corresponds to a particular approach and is separated by a large space. The four bars are placed in increasing order of feature set sizes. In each set of four bars, the leftmost bar corresponds to the least feature set size and the rightmost bar corresponds to the highest feature set size. We may note in Fig 5, that as we increase the feature set sizes, the positive and negative regions grow in size while the boundary regions shrinks. According to the definition of generality in Eq (27), the union of the positive and negative regions represents the generality. This figure highlights the same fact, noted earlier in the previous discussion, i.e., we are able to make more classification decisions for proteins as the level of information increases (or number of features increases). Please be noted that in probabilistic rough sets, it is not always necessary that the addition of features will increase the positive and negative regions. However, we want to emphasize the fact that it will result in improvement in the quality of the regions.

**Fig 6:**
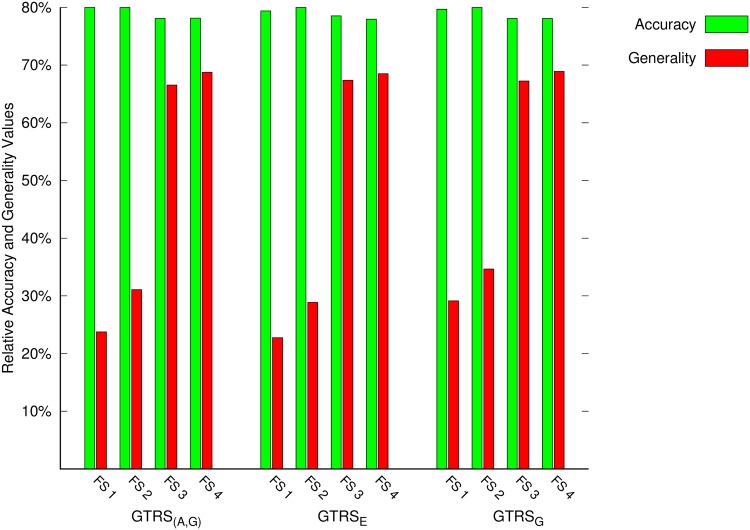
Accuracy and generality results of the GTRS based approaches.

**Fig 7:**
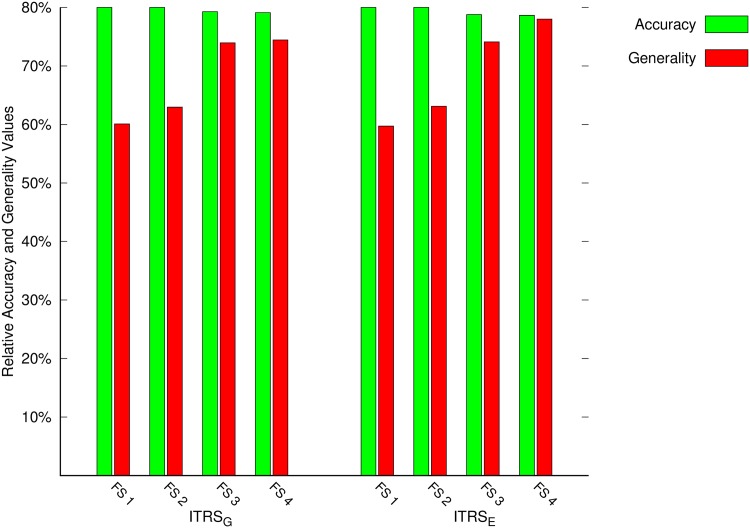
Accuracy and generality results of the ITRS based approaches.

Figs 6 and 7 summarizes the results of accuracy and generality for the considered approaches. The green colour in these figures represent the accuracy and the red colour indicate the generality. The values of accuracy and generality are reported for all four feature sets described in the previous section. It may be noted that for all the approaches, the generality improves as we use higher number of features. However, on the other hand the accuracy is not affected significantly. This means that by increasing the features, we are able to improve the generality while maintaining the similar level of accuracy.

### Comparison with other approaches

In this section, we compare our method to the most widely used group of function prediction techniques. The first choice of comparison is a most recent function prediction tool, namely INGA (Interaction Network GO Annotator) [75], which is a state of the art tool to predict protein functions. INGA is based on a consensus strategy that maximizes the F-score by utilizing protein interaction networks, sequence similarity as well as domain information for protein function prediction. Since, information such as protein domains, protein interaction networks, sequence similarity are directly related to protein function and are at the heart of overwhelming majority of methods that integrate it for protein function prediction, we chose to compare our method against this function prediction tool.

To attain the most accurate comparative results, we run our algorithm for wider range of target classes of the molecular function ontology in a ten-fold cross-validation setting. We present the comparative results (i.e., Accuracy and Generality as defined in Eq (26)) of INGA and our method in Table 5 when tried on *Saccharomyces cerevisiae* proteins. Clearly, our method outperforms IGNA in both aspects. The strength of our method mainly comes from the fact that it is able to defer instances (i.e., proteins) for which there is less characterization evidence at present, thus improving prediction accuracy.

**Table 5:** Comparison of the proposed three way classification method with top performing methods of the field. The target classes comprise of broader gene ontology terms for *Saccharomyces cerevisiae* species proteins.

Method’s Name	Generality	Accuracy
Three way decision using GTRS	68%	78.40%
**Three way decision using ITRS**	**74**%	**79.2**%
INGA (Interaction Network GO Annotator) tool [75]	60%	57%
Jones-UCL [76]	62%	59.5%
Argot [76]	61%	59.4%
BLAST Annotation Transfer (baseline method) [76]	78%	38%

In order to get further insight into the relative performance, we also consider some results that were reported on similar problem. The most recent and well known schemes proposed for the prediction of protein functions are evaluated in the CAFA (Critical Assessment of protein Function Annotation) challenge [76]. The CAFA challenge is conducted after every two years to have comparative evaluation of top schemes for the prediction of protein functions. One of the best sequence alignment algorithm (i.e., BLAST), which is also used as a baseline scheme for annotation transfer, achieved an accuracy of 38% during the CAFA challenge, when tried on molecular function category of GO [76]. Likewise, the top schemes of the challenge have reported to have an accuracy of 59.5% and 59.4%, respectively [76], when tested against heterogeneous ontology classes. On the other hand, our method have achieved an overall accuracy of 80% when tried for the same target classes, depicting a significant gain in terms of prediction accuracy.

Another method, that is more recently proposed, by Mitrofanova et al. in [8], combines inter-species homology data for protein function prediction and reported an accuracy of 97.7% when tried on *Saccharomyces cerevisiae* proteins. However, the results of this method cannot be directly compared with our approach for a number of reasons. Firstly, this method operates on fixed ontology sizes thus giving results for only 16 GO terms (target classes) out of more than 30,000 GO terms. Secondly, the fixed GO terms chosen by the authors limit pertinence of their method to proteins directly annotated to those GO terms, hence limiting the applicability of their algorithm to only a small number of proteins (hence, significant reduction in generality). On the other hand, our algorithm has much wider GO coverage and results presented include all the yeast proteins.

As a final remark, it is pertinent to mention here that although three way classification achieved far better results than the earlier proposed schemes but the main purpose of this study was not the optimization of performance (in terms of precision or accuracy) of the earlier schemes. But rather it should be looked at from the perspective of an examination, feasibility and appropriateness of considering three way classification schemes based on evolving biological information for the task of protein function predictions.

In conclusion, the three way approaches considered in this study achieve an average accuracy of 80%. The incorporation of future information is useful as it improves the generality or applicability of the models while maintaining similar level of accuracy. In particular, there is an increase in generality by more than 40% for the GTRS based approaches and more than 14% for the ITRS based approaches. From the general trend in the results, it is suggested that as more information becomes available, the generality may improve further. These results advocate for the use of three-way approaches for protein functions classification.

## Conclusion

Proteins are involved in almost every biological phenomena and the precise knowledge of their functions plays an essential role in understanding biological processes. Intelligent mechanisms are generally employed to assign and predict functions of proteins. The technological advancements are continuously resulting in new information and features describing protein functions which in turn can be utilized for improving the quality of protein function predictions. An important issue in this context is to develop effective classification schemes and models for classifying protein functions by incorporating evolving information. We propose a three-way decision making approach to address this issue. The approach includes a deferment decision option which is practiced in situations characterized by insufficient and incomplete information. In particular, we considered probabilistic rough sets based models i.e., game-theoretic rough sets and information-theoretic rough sets for inducing and making three-way decisions. An architecture of protein function classification with three-way decisions is also proposed and explained. Experimental results on dataset from *Uniprot* database indicate that as the level of biological information increases, the number of deferred cases are reduced while maintaining similar level of accuracy. In particular, an average accuracy of 80% (±%2) was reported for the considered approaches with an average generality improvement of 33% (±%5) as we increase features.

We investigated the probabilistic rough sets which is one possible way for inducing three-way decisions. Other approaches such as shadowed sets, statistical testing, interval sets and ortho-pairs may also be examined to investigate the potential benefits of three-way approach to protein function classification. Moreover, the three-way approach for protein function classification may further be evaluated and extended by incorporating new features resulting from next generation sequencing data or from other high throughput experiments.

## Supporting information

S1 File “S1_File.zip”. The code (Python/Bash/Matlab) and data files along with instructions are provided as a zip file.
(ZIP)
